# Crystal and mol­ecular structures of *fac*-[Re(Bid)(PPh_3_)(CO)_3_] [Bid is tropolone (TropH) and tri­bromo­tropolone (TropBr_3_H)]

**DOI:** 10.1107/S205322962200465X

**Published:** 2022-05-17

**Authors:** Marietjie Schutte-Smith, Hendrik Gideon Visser

**Affiliations:** a Department of Chemistry, University of the Free State, PO Box 339, Bloemfontein 9301, South Africa

**Keywords:** tropolone, tri­phenyl­phosphane, crystal structure, solid-state NMR spectroscopy, Hirshfeld analysis

## Abstract

Two rhenium com­plexes, namely, *fac*-tricarbon­yl(tri­phenyl­phos­phane-κ*P*)(trop­olonato-κ^2^ *O*,*O*′)rhenium(I), [Re(C_7_H_5_O_2_)(C_18_H_15_P)(CO)_3_] or *fac*-[Re(Trop)(PPh_3_)(CO)_3_] (**1**), and *fac*-tricarbon­yl(3,5,7-tri­bromo­tropolonato-κ^2^ *O*,*O*′)(tri­phenyl­phos­phane-κ*P*)rhenium(I), [Re(C_7_H_2_Br_3_O_2_)(C_18_H_15_P)(CO)_3_] or *fac*-[Re(TropBr_3_)(PPh_3_)(CO)_3_] (**2**) (TropH is tropolone and and TropBr_3_H is tri­bromo­tropolone), were synthesized and their crystal and mol­ecular structures confirmed by single-crystal X-ray diffraction. Both crystallized in the space group *P*  and display an array of inter- and intra­molecular inter­actions which were confirmed by solid-state ^13^C NMR spectroscopy using cross polarization magic angle spinning (CPMAS) techniques, as well as Hirshfeld surface analysis. The slightly longer Re—P distance of **1** [2.4987 (5) *versus* 2.4799 (11) Å for **1** and **2**, respectively] suggests stronger back donation from the carbonyl groups in the former case, possibly due to the stronger electron-donating ability of the unsubstituted trop­o­lon­ate ring system. However, this is not supported in the Re—CO bond distances of **1** and **2**.

## Abstract 2

The crystal structures of two rhenium(I) com­plexes were determined and inter- and intra­molecular inter­actions were confirmed with solid-state NMR spectroscopy.

## Introduction

Rhenium(I) tricarbonyl com­plexes not only have application as models for similar technetium(I) com­plexes in radiophar­macy, but their anti­cancer and anti­microbial properties have recently been investigated by several research groups, including ours (Gantsho *et al.*, 2020 ▸; Collery *et al.*, 2019 ▸; Li *et al.*, 2012 ▸; Otero *et al.*, 2019 ▸; Leonidova & Gasser, 2014 ▸; Brink *et al.*, 2018 ▸; Sovari *et al.*, 2020 ▸, 2021 ▸; Varma *et al.*, 2020 ▸). Similarly, the bidentate ligand tropolone and its derivatives have shown anti­cancer and anti­viral properties on their own (Ishihara *et al.*, 2010 ▸; Borowski *et al.*, 2007 ▸; Dittes *et al.*, 1995*a* ▸) and when combined with metal com­plexes (Ishihara *et al.*, 2010 ▸; Borowski *et al.*, 2007 ▸; Dittes *et al.*, 1995*a* ▸,*b* ▸; Trust, 1975 ▸). Kinetic studies have shown that tropolone and other *O*,*O*′-bidentate ligands like 3-hy­droxy­flavone increase the rate of substitution of water or methanol in *fac*-[Re(Bid)*X*(CO)_3_]^*n*^ (Bid = bidentate ligand, *X* = H_2_O or methanol, and *n* = 0 or 1+) type com­plexes by up to 20000 times (Gantsho *et al.*, 2020 ▸; Schutte *et al.*, 2012 ▸; Schutte-Smith *et al.*, 2019*b* ▸; Schutte *et al.*, 2011 ▸; Manicum *et al.*, 2020 ▸; Schutte-Smith & Visser, 2015 ▸). This kind of mechanistic information is extremely important when designing mol­ecules for anti­cancer, anti­bacterial and anti­viral applications, as well as in radiopharmacy (Collery *et al.*, 2019 ▸; Schutte-Smith *et al.*, 2019*b* ▸). Recently, we showed that kinetic data can be correlated with cytotoxicity and cell availability (Schutte-Smith *et al.*, 2020 ▸).

Our focus is to try to understand the basic chemistry (mechanism of action, structure–activity relationships and stability) of these organometallic com­pounds to aid in the design of new bioactive pharmaceuticals. This also includes the characterization by means of solid- and solution-state multinuclear NMR spectroscopy, single-crystal X-ray diffraction and other spectroscopic methods. The application of solid-state NMR spec­troscopy to study hydrogen-bond and other intra- and inter­molecular inter­actions is growing rapidly, with many research groups involved in the development of new techniques to study crystalline and even amorphous phases, making it a useful tool for our purposes as well (Chierotti & Gobetto, 2008 ▸; Traer *et al.*, 2007 ▸; Zhao *et al.*, 2001 ▸; Schutte-Smith *et al.*, 2019*a* ▸; Wilhelm *et al.*, 2022 ▸).

We report here the crystal and mol­ecular structures of *fac*-[Re(Trop)(PPh_3_)(CO)_3_] (**1**) and *fac*-[Re(TropBr_3_)(PPh_3_)(CO)_3_] (**2**) (TropH is tropolone and TropBr_3_H is tri­bromo­tropolone), together with the solid- and solution-state multinuclear NMR spectroscopic analysis, and we attempt to correlate the spectral data with bond lengths and inter­actions.

## Experimental

### Materials and methods

All reagents employed in the preparation and characterization of the title com­pounds were of analytical grade, were purchased from Sigma–Aldrich or Merck (South Africa) and were used without any further purification; all experiments were performed aerobically. The IR spectra were recorded at room temperature on a PerkinElmer BX II IR spectrometer in the range 4000–370 cm^−1^.

The liquid-state ^1^H, ^13^C and ^31^P NMR spectra were re­cor­ded at 25.0 °C on a 300 MHz Bruker Fourier NMR spectrometer, a 400 MHz Avance III NMR spectrometer and a 600 MHz Avance II Bruker spectrometer, respectively, and methanol-*d*_4_, toluene-*d*_6_ and acetone-*d*_6_ were used as solvents. The chemical shifts (δ) are reported in parts per million (ppm); for methanol-*d*_4_ and acetone-*d*_6_, the spectra were referenced relative to the solvent peak (3.31 ppm for ^1^H and 49.15 ppm for ^13^C, and 2.05 for ^1^H and 29.92 for ^13^C, respectively). Coupling constants (*J*) are reported in Hz. The solid-state NMR spectra were collected on a 400 MHz Bruker Avance III spectrometer equipped with a 4 mm VTN multinuclear double resonance magic angle spinning probe, operating at 25.0 °C. The ^13^C NMR spectra were recorded at 100.6 MHz, using the cross polarization magic angle spinning (CP/MAS) technique. A rotating speed of 10000 Hz was used with a contact time of 2 ms, a recycle delay of 5 s and an acquisition time of 33.9 ms. All the spectra were recorded with 3k scans. The samples were packed in 4 mm zirconia rotors.

### Synthesis and crystallization

#### *fac*-[Re(Trop)(PPh_3_)(CO)_3_] (1)

*fac*-[Re(Trop)(CO)_3_(H_2_O)] (50 mg, 0.122 mmol), synthesized according to a previously reported procedure (Schutte *et al.*, 2012 ▸), was dissolved in acetone (30 ml) and tri­phenyl­phos­phane (32 mg, 0.122 mmol) was added to the solution. The mixture was stirred overnight at room temperature and left to crystallize from the acetone solution (yield: 69 mg, 87%).

IR (KBr, cm^−1^): ν_CO_ = 2010, 1934, 1887. ^1^H NMR (400.13 MHz, acetone-*d*_6_): δ 7.42 (*m*, 15H), 7.23 (*t*, 2H, *J* = 10.6 Hz), 6.91 (*d*, 2H, *J* = 10.8 Hz), 6.84 (*t*, 1H, *J* = 9.6 Hz). ^13^C NMR (100.61 MHz, acetone-*d_6_*): δ 184 (Trop), 138 (Trop), 134 (PPh_3_), 131 (PPh_3_), 129 (PPh_3_), 127 (Trop). ^13^C CP/MAS NMR (100.61 MHz): δ 183, 182, 138, 136, 135, 133, 132, 130, 129, 128, 127, 126. ^31^P (161.97 MHz, acetone-*d*_6_): δ 18.2. Analysis calculated (%): C 51.45, H 3.08, P 4.74; found: C 51.43, H 3.11, P 1.76.

#### *fac*-[Re(TropBr_3_)(PPh_3_)(CO)_3_] (2)

*fac*-[Re(TropBr_3_)(CO)_3_(H_2_O)] (50 mg, 0.077 mmol), synthesized according to a previously reported procedure (Schutte *et al.*, 2008 ▸), was dissolved in acetone (30 ml) and tri­phenyl­phos­phane (20 mg, 0.0077 mmol) was added to the solution. The mixture was stirred overnight at room temperature and left to crystallize from the acetone solution (yield: 62.5 mg, 91%).

IR (KBr, cm^−1^): ν_CO_ = 2018, 1922, 1889. ^1^H NMR (400.13 MHz, acetone-*d*_6_): δ 8.15 (s, 2H), 7.42 (m, 15H). ^13^C NMR (150.95 MHz, acetone-*d*_6_): δ 176 (Trop), 143 (Trop), 134 (PPh_3_), 132 (Trop), 129 (Trop). ^13^C CP/MAS NMR (100.61 MHz): δ 135, 133, 131, 129. ^31^P (161.97 MHz, acetone-*d*_6_): δ 19.6. Analysis calculated (%): C 37.77, H 1.92, P 3.48; found: C 37.81, H 1.90, P 3.45.

### Refinement

Crystal data, data collection and structure refinement details are summarized in Table 1 ▸. All aromatic H atoms were placed in geometrically idealized positions (C—H = 0.95 Å) and constrained to ride on their parent atoms, with *U*_iso_(H) = 1.2*U*_eq_(C).

## Results and discussion

### Synthesis

*fac*-[Re(Trop)(PPh_3_)(CO)_3_] (**1**) and *fac*-[Re(TropBr_3_)(PPh_3_)(CO)_3_] (**2**) were synthesized from the respective aqua com­plexes, which were synthesized according to previously reported procedures (Gantsho *et al.*, 2020 ▸; Schutte *et al.*, 2008 ▸, 2012 ▸). The synthesis of **1** was described previously, but crystals suitable for single-crystal X-ray diffraction could not be obtained at the time (Gantsho *et al.*, 2020 ▸). Compounds **1** and **2** were synthesized in good yield from acetone solutions, after stirring the respective aqua com­plexes with one equivalent of tri­phenyl­phoshane overnight.

In the ^1^H NMR spectra, a significant downfield shift is observed from **1** (7.23, 6.91 and 6.84 ppm) to **2** (8.15 ppm) for the tropolonate and tri­bromo­tropolonate H atoms, respectively, which is expected due to the electron-withdrawing Br atoms in **2** causing deshielding of the nuclei. This is confirmed in the ^31^P NMR spectra with a slight downfield shift in the phospho­rus peak of **1** at 18.2 ppm and **2** at 19.6 ppm. The IR carbonyl stretching frequencies of **1** (2010, 1934 and 1887 cm^−1^) are lower than **2** (2018, 1922 and 1889 cm^−1^), which is expected since the tropolonate ligand in **1** is more electron donating than the tri­bromo­tropolonate ligand in **2**, therefore implying stronger backbonding from the carbonyl ligands to the metal centre and resulting in lower CO stretching frequencies. This, in turn, labilizes the phos­phane ligand in the sixth position and is confirmed in the solid-state structures, with the Re—P bond lengths reported as 2.4987 (5) Å for **1** and 2.4799 (11) Å for **2**.

### X-ray crystallography

**Figure 1:**
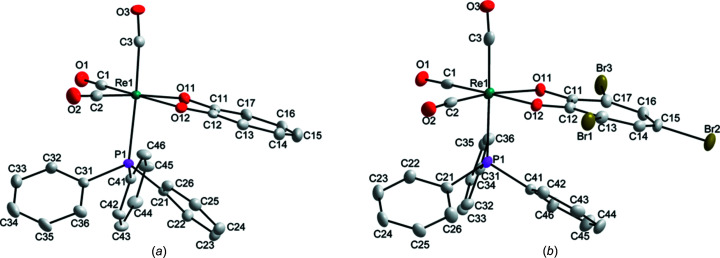
The mol­ecular structures of (*a*) *fac*-[Re(Trop)(PPh_3_)(CO)_3_] (**1**) and (*b*) *fac*-[Re(TropBr_3_)(PPh_3_)(CO)_3_] (**2**), showing the atom-numbering schemes. H atoms have been omitted for clarity. Displacement ellipsoids are drawn at the 50% probability level.

A summary of the crystal data for **1** and **2** is given in Table 1 ▸. *fac*-[Re(Trop)(PPh_3_)(CO)_3_], **1**, crystallized in the triclinic space group *P* 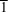 with one mol­ecule in the asymmetric unit. The mol­ecular diagram and selected bond lengths and angles are given in Fig. 1 ▸ and Table 2 ▸, respectively. Three inter­molecular and one intra­molecular hydrogen-bonding inter­action (C—H⋯O) are observed in the structure, as well as two inter­molecular C—O⋯π and one intra­molecular π–π inter­action (Figs. S1 and S2 in the supporting information). A summary of the geometric parameters of these inter­actions is given in Tables S1 and S2 in the supporting information. Inter­estingly, the hydrogen-bond inter­actions involve the tropolonate ligand and the C atoms of the C41-ring as C—H donor atoms, and the O atoms of the tropolonate ring and the O atom of a carbonyl ligand as acceptor atoms. The π-inter­actions, on the other hand, involve inter­actions between the carbonyl O2 and O3 atoms and the centroids of the five-membered Re/O11/C11/C12/O12 ring, as well as the arene rings of the phos­phane ligand (C21–C26 and C31–C36).

*fac*-[Re(TropBr_3_)(PPh_3_)(CO)_3_], **2**, also crystallized in the triclinic space group *P* 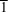 with one mol­ecule in the asymmetric unit. The mol­ecular diagram is given in Fig. 1 ▸ and selected bond lengths and angles are provided in Table 3 ▸. Four inter­molecular hydrogen-bond inter­actions (three C—H⋯O and one C—H⋯Br) and one intra­molecular hydrogen-bond inter­action (C—H⋯O) are observed in the structure of **2** (Fig. S3 in the supporting information). A short contact of 3.250 (4) Å is observed between Br2 and O3(−*x* + 1, −*y* + 1, −*z* + 1) (Fig. S4). Two inter­molecular contacts form an infinite one-dimensional chain with base vector [110] between Br1 and Br3(*x* − 1, *y* − 1, *z*), and between Br3 and Br1(*x* + 1, *y* + 1, *z*), both with a distance of 3.4809 (7) Å (Fig. S5). A range of π-inter­actions are observed: one *X*—H⋯π, two π–π and three *Y*—*X*⋯π inter­actions ranging between 3.438 (4) and 3.865 (2) Å (Fig. S4). A summary of the geometric parameters of these inter­actions is given in Tables S3 and S4 in the supporting information. All three Br atoms are involved in short contacts, while Br2 is additionally involved in a π-inter­action and Br3 is involved as an acceptor in a hydrogen-bond inter­action. All five of the ring systems, *i.e.* the three arene rings of the PPh_3_ ligand, the tropolonate ring and the Re1/O11/C11/C12/O12 five-membered ring, are involved in the π-inter­actions.

The bond lengths and angles of **1** and **2** com­pare well with each other and also with similar structures in the literature (Gantsho *et al.*, 2020 ▸; Schutte-Smith *et al.*, 2019*b* ▸; Schutte *et al.*, 2007 ▸, 2008 ▸; Manicum *et al.*, 2020 ▸; Bochkova *et al.*, 1987 ▸; Kydonaki *et al.*, 2016 ▸). The Re—P1 bond length of **1** is slightly longer than in **2**, possibly due to the electron-withdrawing effect of the three Br atoms on the backbone of **2**. The tropolonate ligand in **1** donates more electron density to the rhenium metal centre, initiating more backbonding from the carbonyl ligands, labilizing the Re—P bond. Although this is what we expect, it is not observed in the Re—CO bond lengths of **1** and **2**, which do not differ significantly. Considering the angles around the Re^I^ metal core, a good correlation between **1** and **2** is found. The small bite angles of 73.99 (5) and 73.05 (10)° for **1** and **2**, respectively, indicate the degree of distortion of the octa­hedral geometry, which is normal and within the range of other similar structures where a five-membered *O*,*O*′-chelate ring is formed with the metal centre (Gantsho *et al.*, 2020 ▸; Schutte *et al.*, 2007 ▸, 2008 ▸; Schutte-Smith *et al.*, 2019*b* ▸; Bochkova *et al.*, 1987 ▸). In the case of a six-membered *O*,*O*′-chelate ring (with PPh_3_ in the sixth position), the bite angle is slightly larger, with values ranging between 82.2 and 84.7° (Manicum *et al.*, 2020 ▸; Kydonaki *et al.*, 2016 ▸).

The tropolonate and tri­bromo­tropolonate ligands bend slightly towards the tri­phenyl­phos­phane ligand in **1** and **2**, with dihedral angles between the plane through the Re(CO)_3_ entity and the ligand (the plane through Re/C1/O1/C2/O2 and the plane through O11/O12/C11–C17) of 8.85 (8) and 12.43 (14)°, respectively (illustrated in Fig. S6 in the supporting information). In **2**, the Br atoms are slightly ‘out of plane’ with respect to the tropolonate ring (C11–C17) at −0.1463 (4), 0.1760 (5) and −0.2114 (5) Å for Br1, Br2 and Br3, respectively. This could be due to the different inter­actions observed: the inter­molecular contacts between Br1 and Br3 and the C—H⋯Br3 hydrogen-bond inter­action, and the Br2⋯O3 short contact and C15—Br2⋯*Cg*1(−*x* + 1, −*y* + 1, −*z* + 1) π-inter­action for Br2 (*Cg*1 is the centroid of the Re/C1/O1/C2/O2 ring).

**Figure 2:**
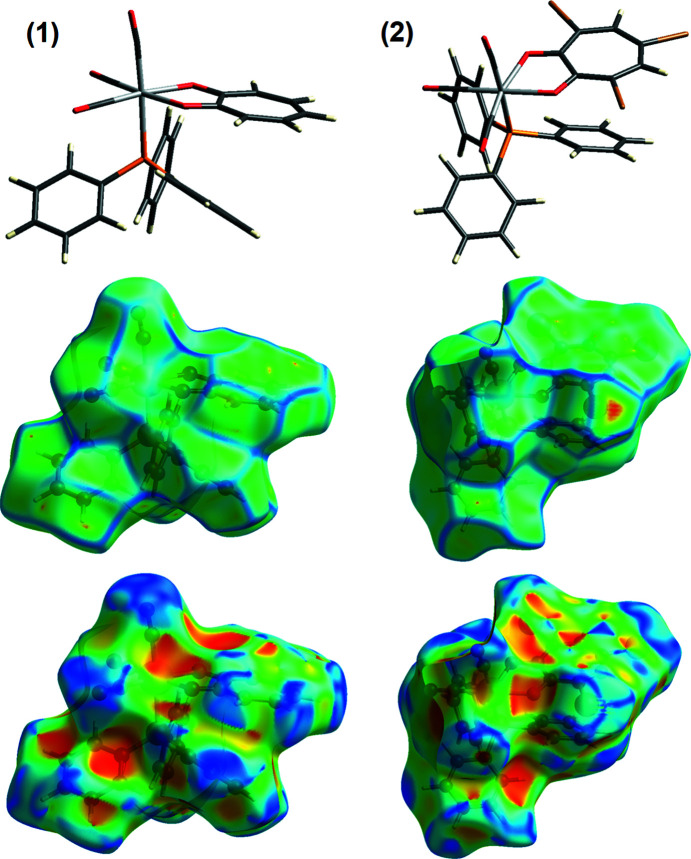
The Hirshfeld surfaces of **1** and **2**, illustrating a curvedness plot (middle), a shape index plot (bottom) and the mol­ecular diagram for clarity of **1** and **2**.

The Hirshfeld surfaces of **1** and **2** are illustrated in Fig. 2 ▸ (Spackman & Jayatilaka, 2009 ▸). The mol­ecular diagram of the com­pound is given at the top of the figure to illustrate the orientation of each com­pound in the curvedness (middle) and shape index (bottom) plots below it. In **1**, the blue concave regions around O2 and O3 correspond to the C2—O2⋯*Cg*3(−*x* + 2, −*y*, −*z* + 1) (*Cg*3 is the centroid of the C31–C36 ring) and C3—O3⋯*Cg*1(−*x* + 2, −*y*, −*z*) *Y*—*X*⋯π inter­actions as given in Table S2 (see supporting information), while a red convex region around O1 corresponds to the C17—H17⋯O1(−*x* + 2, −*y* + 1, −*z*) hydrogen-bond inter­action (Table S1). The large red convex area above the rhenium five-membered ring and atoms O11 and O12 correlates with the three hydrogen-bond inter­actions C44—H44⋯O12(*x*, *y* + 1, *z*), C45—H45⋯O11(−*x* + 2, −*y* + 1, −*z*) and C46—H46⋯O11, and the π-inter­action C3—O3⋯*Cg*1(−*x* + 2, −*y*, −*z*) (Table S1 and Table S2).

In **2**, the blue and red adjacent triangles above the tri­bromo­tropolonate ring system correlate with the π–π inter­actions given in Table S4 (Seth *et al.*, 2011 ▸). Blue convex regions are observed around the donor atoms Br1, Br2 and Br3 [C15—Br2⋯*Cg*1(−*x* + 1, −*y* + 1, −*z* + 1), Br1⋯Br3(*x* − 1, *y* − 1, *z*), Br3⋯Br1(*x* + 1, *y* + 1, *z*) and Br2⋯O3(−*x* + 1, −*y* + 1, −*z* + 1)], as well as red concave regions above the five-membered rhenium ring system [C15—Br2⋯*Cg*1(−*x* + 1, −*y* + 1, −*z* + 1) and C36—H36⋯O11] and atom O3 [Br2⋯O3(−*x* + 1, −*y* + 1, −*z* + 1) and C46—H46⋯O3(*x* + 1, *y*, *z*)] which correlates with the data given in Tables S3 and S4 in the supporting information.

Overall, the curvedness of **1** has less ‘flat’ regions com­pared to **2**, and com­pares well with the increased number of π-inter­actions observed in **2** com­pared to **1**.

**Figure 3:**
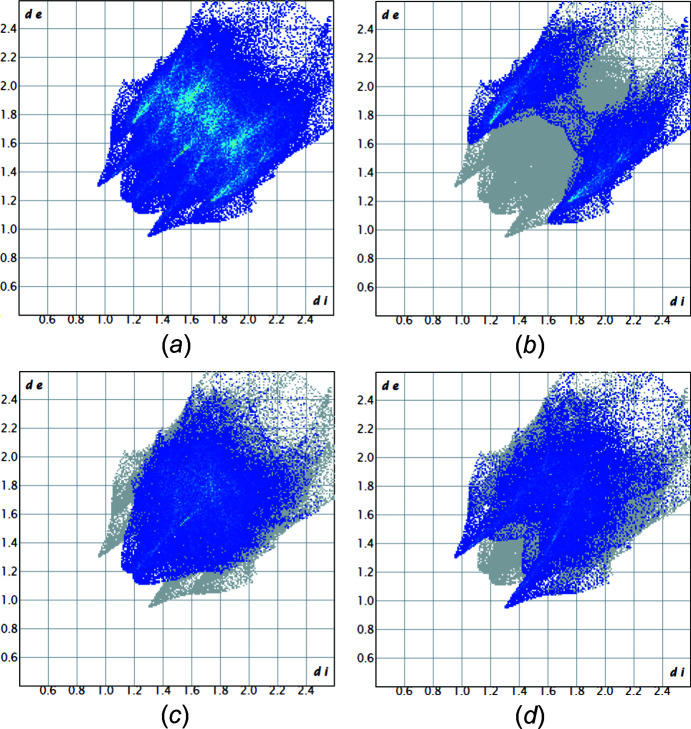
Fingerprint plots of **1**: (*a*) full plot with the total Hirshfeld surface area of the mol­ecules. Fingerprint plots of **1** resolved into (*b*) H⋯C/C⋯H (26.5%), (*c*) H⋯H (38.1%) and (*d*) O⋯H/H⋯O (28%).

Figs. 3 ▸ and 4 ▸ show the fingerprint plots of **1** and **2**, respectively. Fingerprint plots can be decom­posed to separate the contributions from different types of inter­actions that overlap in the full fingerprint. In **1**, the proportion of O⋯H/H⋯O inter­­actions com­prise 28.1%, H⋯H com­prise 38.1% and H⋯C/C⋯H com­prise 26.5% of the total Hirshfeld surfaces for each mol­ecule. In **2**, the distribution is slightly different; the C⋯H/H⋯C inter­actions com­prise 16%, the Br⋯H/H⋯Br inter­actions com­prise 15.2%, the Br⋯Br inter­actions com­prise 5.1%, the H⋯H inter­actions com­prise 25% and the O⋯H/H⋯O inter­actions com­prise 23.7% of the total Hirshfeld surfaces.

When *d*_norm_ (as defined and explained by Spackman & Jayatilaka, 2009 ▸) is mapped on a Hirshfeld surface, inter­molecular contacts appear as red spots, contacts shorter than van der Waal separations, on a largely blue surface. It has been proven to be useful as an unbiased method to identify close inter­molecular contacts, even in com­plex crystal structures.

**Figure 5:**
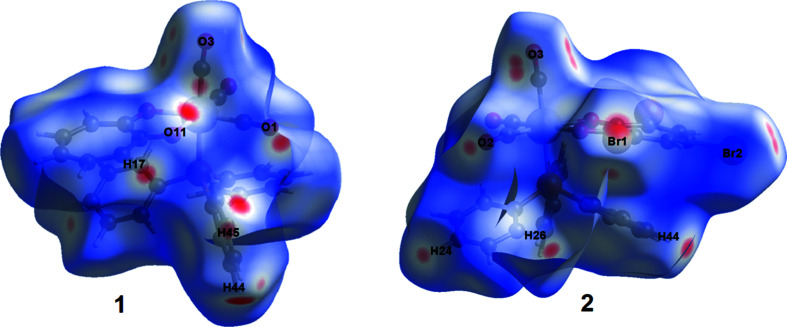
The Hirshfeld surfaces for **1** and **2** mapped with *d*_norm_ over the range from −0.2 to 1.4.

*d*_norm_ Hirshfeld plots of **1** and **2** are presented in Fig. 5 ▸, indicating the red spots associated with close contacts. Not all inter­actions are shown for conciseness because all the inter­actions are not visible from one orientation. All the inter­actions reported in Tables S1–S4 correlate with these plots.

By com­paring **1** and the previously reported bis­(tri­phenyl­phos­phane) com­plex [Re(Trop)(PPh_3_)_2_(CO)_2_] (**3**) (Gantsho *et al.*, 2020 ▸), it is clear that most of the bond lengths around the metal centre change when the axial carbonyl ligand is substituted by a second PPh_3_ ligand (Table 4 ▸). When the carbonyl ligand is substituted by a PPh_3_ ligand, more electron density is donated to the Re^I^ metal centre, shortening the equatorial Re—CO bond lengths from 1.900 (2) and 1.912 (2) Å to 1.883 (3) and 1.887 (3) Å. PPh_3_ also has a weaker *trans* effect than CO, which is evident in the shortening of the Re1—P1 bond(s).

Inter­estingly, the *trans* effect is clearly observed in the axial Re—CO distances in the solid-state crystal structures of *fac*-[Re(Trop)(CO)_3_(H_2_O)] (Schutte *et al.*, 2012 ▸), *fac*-[Re(Trop)(Py)(CO)_3_] (Schutte *et al.*, 2012 ▸) and *fac*-[Re(Trop)(PPh_3_)(CO)_3_] [increasing from 1.890 (7) to 1.919 (4) to 1.944 (2) Å], and also in *fac*-[Re(TropBr_3_)(CO)_3_(H_2_O)] (Schutte *et al.*, 2008 ▸), *fac*-[Re(TropBr_3_)(Br)(CO)_3_]^−^ (Schutte *et al.*, 2007 ▸) and *fac*-[Re(TropBr_3_)(PPh_3_)(CO)_3_] [increasing from 1.882 (7) to 1.897 (3) to 1.950 (5) Å] as the *trans* effect increases according to the following trend: H_2_O < Py < Br < P*R*_3_ < CO (with Py = pyridine and P*R*_3_ = tertiary phos­phane).

### Solid-state NMR

In solid-state ^13^C NMR spectroscopy, the cross polarization magic angle spinning (CP/MAS) technique is often used to enhance the polarization of the low-abundance ^13^C nuclei *via* its inter­action with ^1^H nuclei. The effectiveness of the CP/MAS technique depends on the magnitude of ^1^H–^13^C dipolar coupling (Freitas *et al.*, 2016 ▸; Conte *et al.*, 2004 ▸; Smernik *et al.*, 2002 ▸). It is expected that the observed hydrogen-bond inter­actions, as well as other short contacts and π-inter­actions in the solid state, will deshield the C atoms and cause a downfield shift in the solid-state ^13^C NMR spectra (Patterson-Elenbaum *et al.*, 2006 ▸). In the liquid state, the intra- and inter­molecular inter­actions are disrupted because of the motion of the mol­ecules within the solution; thus, we only observe the dynamic average of the motion. The degree of inter­actions present in the solid-state can be determined by the difference in chemical shift values (Δδ) of the specific C atoms in the liquid- *versus* solid-state NMR spectra (Patterson-Elenbaum *et al.*, 2006 ▸). A larger difference in chemical shift is normally indicative of a stronger inter­action, which is determined by the specific bond length and angle (Siskos *et al.*, 2017 ▸).

It is known that broad peaks (or no peaks) are observed when there are not many C atoms that are directly bound to H atoms (Freitas *et al.*, 2016 ▸), which is the case in **2**. Nevertheless, we aimed to correlate the change in chemical shift from the ^13^C liquid-state NMR to the solid-state ^13^C NMR to the inter­actions observed in the crystal structures.

**Figure 6:**
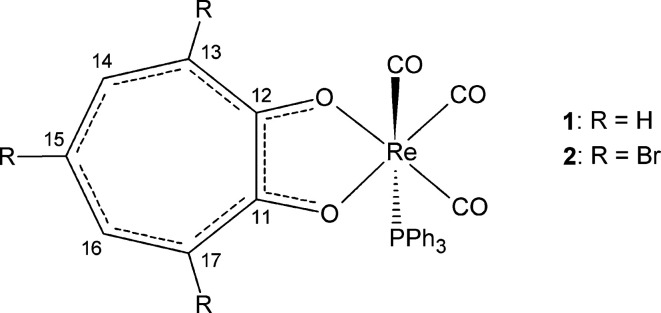
The atom-numbering schemes of **1** and **2**.

**Figure 7:**
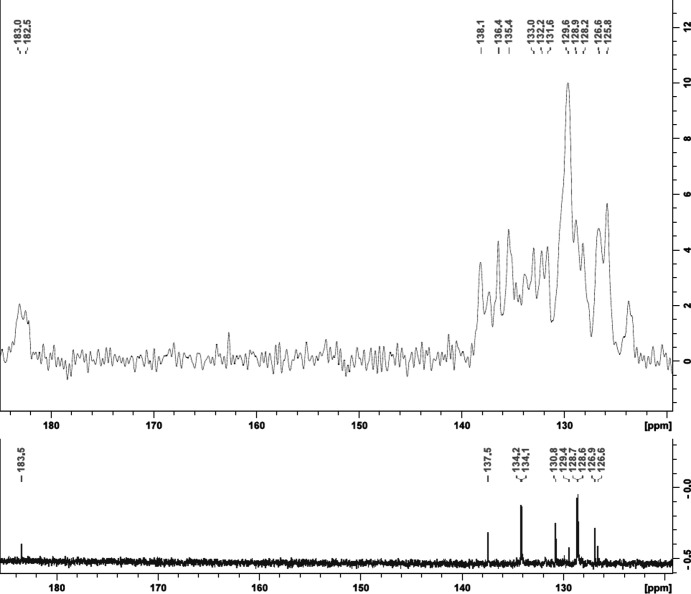
Solid-state *versus* liquid-state ^13^C NMR spectra of **1**.

Fig. 6 ▸ provides the numbering scheme of atoms in **1** and **2**. The solid-state ^13^C NMR data of **1** did not shift much from the solution state to the solid state, with not more than a 1 ppm change (Δδ) in the chemical shift at most, which is basically negligible (Fig. 7 ▸). Four hydrogen-bond inter­actions and three π-inter­actions are observed in **1** (Tables S1 and S2 in the supporting information), two of the π-inter­actions being very weak (distance > 3.8 Å). The five inter­actions that are considered to be stronger with shorter distances involve the PPh_3_ ligand, the O atoms of the tropolonate ligand, the carbonyl ligands and the centroid of the five-membered Re1/O11/C11/C12/O12 ring system. The carbonyl ligands are not visible on the liquid- and solid-state ^13^C NMR spectra due to the economic and time implications involved to observe it. IR spectroscopy are used to confirm the presence of the carbonyl ligands in this type of com­plex.

In the solution-state ^13^C NMR spectra, the peak at 184 ppm is assigned to C11 and C12, and seeing that these atoms are bound to O11 and O12 (involved in three inter­actions) and are part of the five-membered ring system (Re1/O11/C11/C12/O12), a downfield shift is expected because of the effect of the deshielding of these inter­actions. However, it shifted slightly upfield to 183 and 182 ppm, and yielded two peaks in the solid state com­pared to a single peak in the solution state. This is due to the fact that C11 and C12 are not equivalent in the solid state because of the inter­actions observed in the crystal structure: C17—H17⋯O1(−*x* + 2, −*y* + 1, −*z*), C45—H45⋯O11(−*x* + 2, −*y* + 1, −*z*) and C46—H46⋯O11 all indirectly involve C11, while C44—H44⋯O12(*x*, *y* + 1, *z*) is the only inter­action that indirectly involves C12; thus, the splitting of the single peak in the solid state.

The single peak for C13, C14, C16 and C17 at 138 ppm, the peaks for the PPh_3_ ligand at 134, 131 and 129 ppm, and the single peak for C15 at 127 ppm in the solution state are not as well defined in the solid state and yield a broad peak from 138 to 126 ppm, similar to the range found in the solution state; we expected a downfield shift because of the inter­actions involving the tropolonate ring system and one arene ring of the PPh_3_ ligand. We could, however, see some significant splitting of the peaks; com­pared to the five single peaks at 138, 134, 131, 129 and 127 ppm in the solution state, splitting of the peaks (although it is a broad peak) is seen in the solid state, indicating that many of the C atoms are not equivalent anymore because of the inter­actions observed in the crystal structure [C17—H17⋯O1(−*x* + 2, −*y* + 1, −*z*), meaning C13, C14, C16 and C17 are not equivalent anymore; C44—H44⋯O12(*x*, *y* + 1, *z*), C45—H45⋯O11(−*x* + 2, −*y* + 1, −*z*) and C46—H46⋯O11, meaning the C41–C46 arene ring in PPh_3_ is not equivalent to the C21–C26 and C31–C36 arene rings].

**Figure 8:**
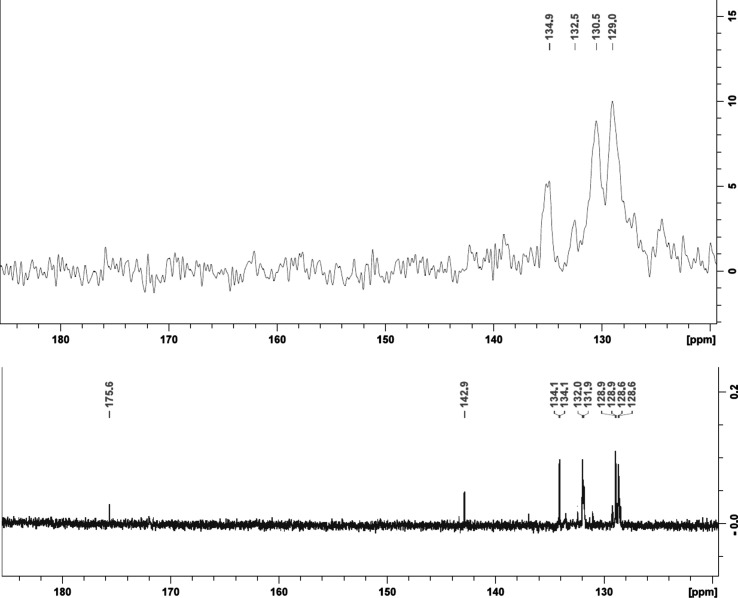
Solution-state *versus* solid-state ^13^C NMR spectra of **2**.

In the case of **2**, the fact that the tri­bromo­tropolonate ring system only has two H atoms directly bound to C atoms had an impact on the solid-state ^13^C NMR spectra and we only observe the PPh_3_ ligand, and the seven C atoms in the tri­bromo­tropolonate ligand are not observed (Fig. 8 ▸) (Freitas *et al.*, 2016 ▸). In the solution-state spectra, the PPh_3_ ligand has a single peak at 134 ppm which split up into four peaks at 135, 132, 131 and 129 ppm in the solid-state spectra. Again, this is because the C atoms are not equivalent in the solid state.

## Conclusion

Two new crystal structures of rhenium(I) tricarbonyl com­plexes with either a tropolonate or a tri­bromo­tropolonate bi­den­tate ligand are reported and correspond well with similar known structures. The solid-state NMR data indicated the presence of inter- and intra­molecular inter­actions, as seen by the splitting of some signals, but unfortunately, due to the fact that both **1** and **2** contain only a few C—H units each, credible chemical shifts could not be obtained and correlated with the crystal data. The inter­molecular inter­actions obtained from *PLATON* (Spek, 2020 ▸) correlate with the Hirshfeld surfaces generated with *CrystalExplorer* (Spackman *et al.*, 2021 ▸).

## Supplementary Material

Crystal structure: contains datablock(s) 1, 2, global. DOI: 10.1107/S205322962200465X/oc3014sup1.cif

Structure factors: contains datablock(s) 1. DOI: 10.1107/S205322962200465X/oc30141sup2.hkl

Structure factors: contains datablock(s) 2. DOI: 10.1107/S205322962200465X/oc30142sup3.hkl

Supporting information file. DOI: 10.1107/S205322962200465X/oc30141sup4.cml

Supporting information file. DOI: 10.1107/S205322962200465X/oc30142sup5.cml

Supporting information file. DOI: 10.1107/S205322962200465X/oc3014sup6.pdf

CCDC references: 2108567, 2108568

## Figures and Tables

**Figure 4:**
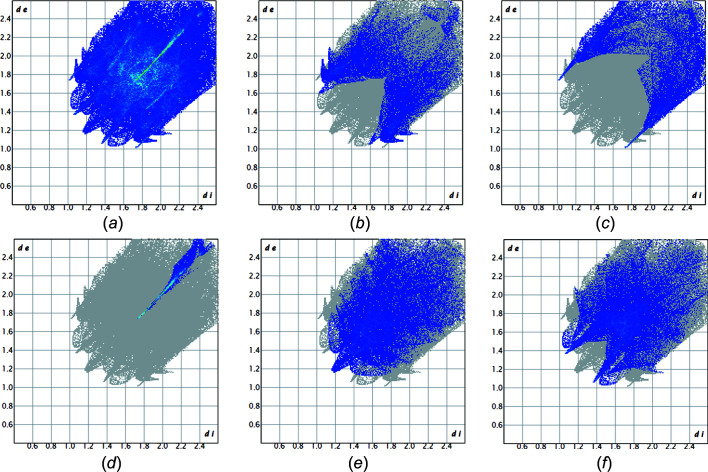
Fingerprint plots of **2**: (*a*) full plot with the total Hirshfeld surface area of the mol­ecules. Fingerprint plots of **2** resolved into (*b*) C⋯H/H⋯C (16%), (*c*) Br⋯H/H⋯Br (15.2%), (*d*) Br⋯Br (5.1%), (*e*) H⋯H (25%) and (*f*) O⋯H/H⋯O (23.7%).

**Table 1:** Experimental details For both structures: triclinic, *P* 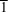 , *Z* = 2. Experiments were carried out with Mo *K*α radiation using a Bruker D8 Quest Eco Chi Photon II CPAD diffractometer for **1** and a Bruker D8 Venture 4K Kappa Photon III C28 diffractometer for **2**. Absorption was corrected for by multi-scan methods (*SADABS*; Bruker, 2012 ▸). H-atom parameters were constrained.

	**1**	**2**
Crystal data		
Chemical formula	[Re(C_7_H_5_O_2_)(C_18_H_15_P)(CO)_3_]	[Re(C_7_H_2_Br_3_O_2_)(C_18_H_15_P)(CO)_3_]
*M*_r_	653.61	890.32
Temperature (K)	100	104
*a*, *b*, *c* (Å)	9.9301 (11), 10.1686 (10), 12.7882 (14)	8.5413 (12), 8.7024 (13), 20.376 (3)
α, β, γ (°)	80.948 (3), 71.899 (3), 88.682 (3)	102.221 (5), 93.891 (5), 109.093 (5)
*V* (Å^3^)	1211.6 (2)	1383.3 (3)
μ (mm^−1^)	5.12	8.82
Crystal size (mm)	0.27 × 0.17 × 0.13	0.18 × 0.04 × 0.04

Data collection		
*T*_min_, *T*_max_	0.357, 0.511	0.690, 0.728
No. of measured, independent and observed [*I* > 2σ(*I*)] reflections	25538, 5820, 5726	34880, 6818, 5913
*R*_int_	0.037	0.068
(sin θ/λ)_max_ (Å^−1^)	0.661	0.668

Refinement		
*R*[*F* ^2^ > 2σ(*F* ^2^)], *wR*(*F* ^2^), *S*	0.015, 0.038, 1.08	0.029, 0.062, 1.06
No. of reflections	5820	6818
No. of parameters	316	343
Δρ_max_, Δρ_min_ (e Å^−3^)	0.46, −0.60	0.93, −1.49

Computer programs: *APEX2* (Bruker, 2012 ▸), *SAINT-Plus* (Bruker, 2012 ▸), *SHELXT* (Sheldrick, 2015*a* ▸), *SHELXL2018* (Sheldrick, 2015*b* ▸), *WinGX* (Farrugia, 2012 ▸) and *DIAMOND* (Brandenburg & Putz, 2019 ▸).

**Table 2:** Selected geometric parameters (Å, °) for **1**

Re1—C1	1.900 (2)	Re1—O12	2.1322 (13)
Re1—C2	1.912 (2)	Re1—O11	2.1345 (13)
Re1—C3	1.944 (2)	Re1—P1	2.4987 (5)
			
O12—Re1—O11	73.99 (5)	O12—Re1—P1	88.10 (4)
C3—Re1—P1	177.15 (6)	O11—Re1—P1	86.59 (4)

**Table 3:** Selected geometric parameters (Å, °) for **2**

Re1—C2	1.903 (4)	Re1—O12	2.127 (3)
Re1—C1	1.917 (4)	Re1—O11	2.159 (2)
Re1—C3	1.950 (5)	Re1—P1	2.4799 (11)
			
O12—Re1—O11	73.05 (10)	C12—O12—Re1	118.7 (2)
C3—Re1—P1	176.09 (11)	C11—O11—Re1	117.4 (2)

**Table 4:** Comparison of bond lengths (Å) of *fac*-[Re(Trop)(PPh_3_)(CO)_3_] (**1**) and [Re(Trop)(PPh_3_)_2_(CO)_2_] (**3**)

Bond	**1**	**3**
Re1—C1	1.900 (2)	1.883 (3)
Re1—C2	1.912 (2)	1.887 (3)
Re1—O11	2.1345 (13)	2.1578 (19)
Re1—O12	2.1322 (13)	2.1548 (18)
Re1—P1	2.4987 (5)	2.4302 (8)
Re1—P2		2.4239 (8)
