# Supplementary material for: Crystal and mol­ecular structures of fac-[Re(Bid)(PPh3)(CO)3] [Bid is tropolone (TropH) and tri­bromo­tropolone (TropBr3H)]
Source: Acta Crystallogr C Struct Chem. 2022 May 17;78(Pt 6):351–9. doi: 10.1107/S205322962200465X (PMC9167630; doi:10.1107/S205322962200465X)
Supplement: Supplementary file 6 [file c-78-00351-sup6.pdf]

STRUCTURAL CHEMISTRY

Volume 78 (2022)

Supporting information for article:

Crystal and molecular structures of *fac*-[Re(Bid)(PPh_3)$(CO)_3$] [Bid is tropolone (TropH) and tribromotropolone ($TropBr_3H$)]

Marietjie Schutte-Smith and Hendrik Gideon Visser

S1. Additional crystallographic figures and tables

Some additional figures and tables of the interactions observed in the structures of **1** and **2** are provided here.

Figure S1 Illustration of the hydrogen bonding interactions observed in the structure of **1** (pink dotted lines). Hydrogen atoms not part of the interactions are omitted for clarity.

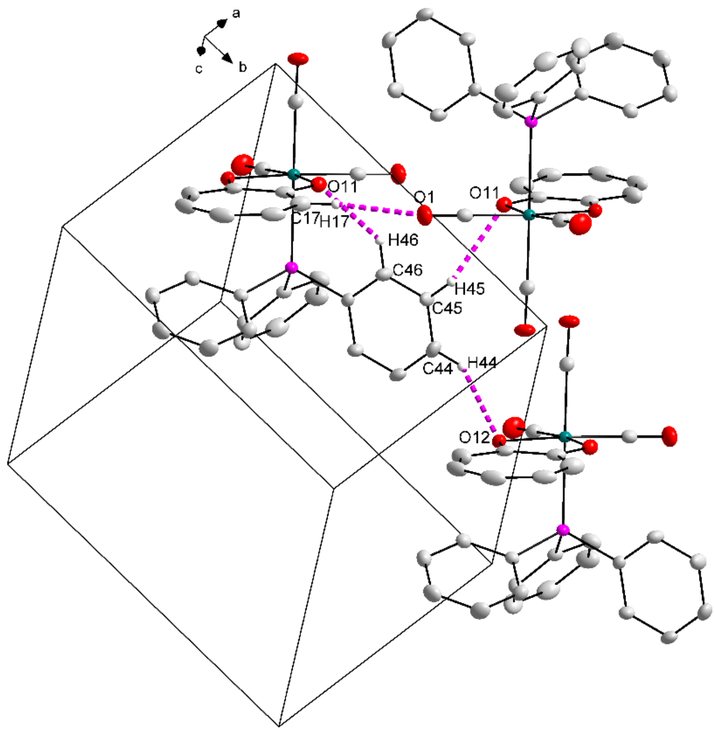

Table S1 Hydrogen bonding interactions observed in the structure of **1**.

D—H···*A*	*D*—H (Å)	H···*A* (Å)	*D*···*A* (Å)	*D*—H···*A* (°)
C17—H17···O1^{i}	0.95	2.54	3.471 (3)	165.7
C44—H44···O12^{ii}	0.95	2.38	3.227 (2)	148.8
C45—H45···O11^{i}	0.95	2.49	3.286 (3)	141.6
C46—H46···O11	0.95	2.31	3.102 (2)	140.9

Symmetry code(s): (i) −*x*+2, −*y*+1, −*z*; (ii) *x*, *y*+1, *z*.

Figure S2 Illustration of the π-interactions observed in the structure of **1** (green dotted lines). Hydrogen atoms omitted for clarity.

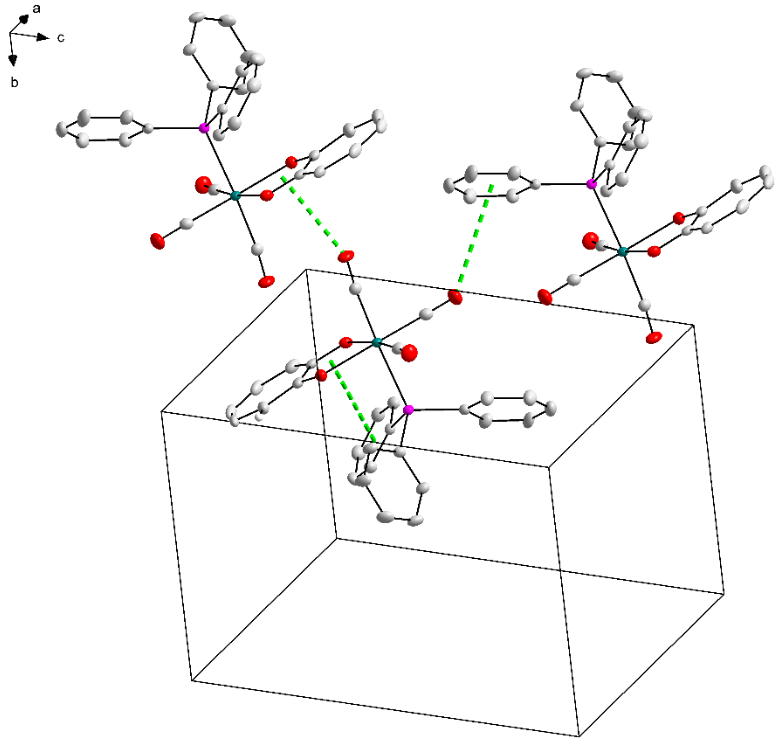

Table S2 Summary of the π-interactions observed in the structure of **1**.

	O···Cg (Å)	Cg···Cg (Å)	Y—X···Cg (°)
C2—O2···Cg3^{i}	3.840(2)		133.81(15)
C3—O3···Cg1^{ii}	3.3241(18)		152.79(15)
Cg1···Cg2		3.9499(12)	

Cg1 = centroid of Re1,O11,C11,C12,O12; Cg2 = centroid of C21-C26; Cg3 = centroid of C31-C36. Symmetry codes: (i) 2−x,-y,1−z, (ii) 2−x,-y,−z.

Figure S3 Hydrogen interactions observed in the structure of **2** (pink dotted lines). Hydrogen atoms not part of the interactions are omitted for clarity.

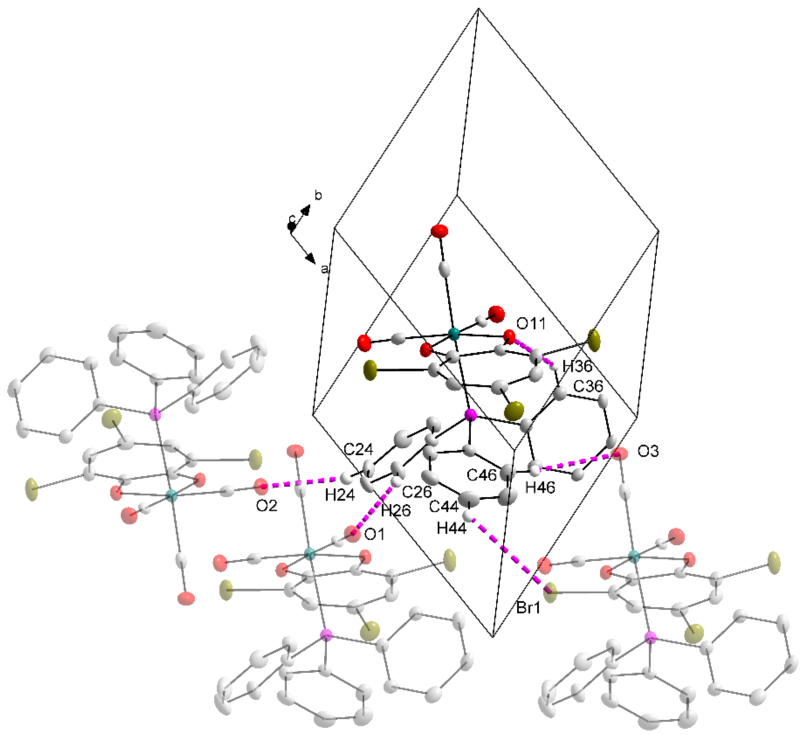

Table S3 Hydrogen bonding interactions observed in the structure of **2**.

D—H···*A*	*D*—H (Å)	H···*A* (Å)	*D*···*A* (Å)	*D*—H···*A* (°)
C24—H24···O2^{i}	0.95	2.58	3.390 (5)	143.5
C26—H26···O1^{ii}	0.95	2.54	3.350 (5)	143.3
C36—H36···O11	0.95	2.52	3.299 (5)	139.2
C44—H44···Br3^{ii}	0.95	2.88	3.819 (4)	168.3
C46—H46···O3^{iii}	0.95	2.58	3.354 (5)	138.4

Symmetry code(s): (i) −*x*, −*y*, −*z*; (ii) *x*, *y*−1, *z*; (iii) *x*+1, *y*, *z*.

Figure S4 Illustration of the π-interactions (green dotted lines) and short contact (blue dotted line) observed in the structure of **2**. Hydrogen atoms are omitted for clarity.

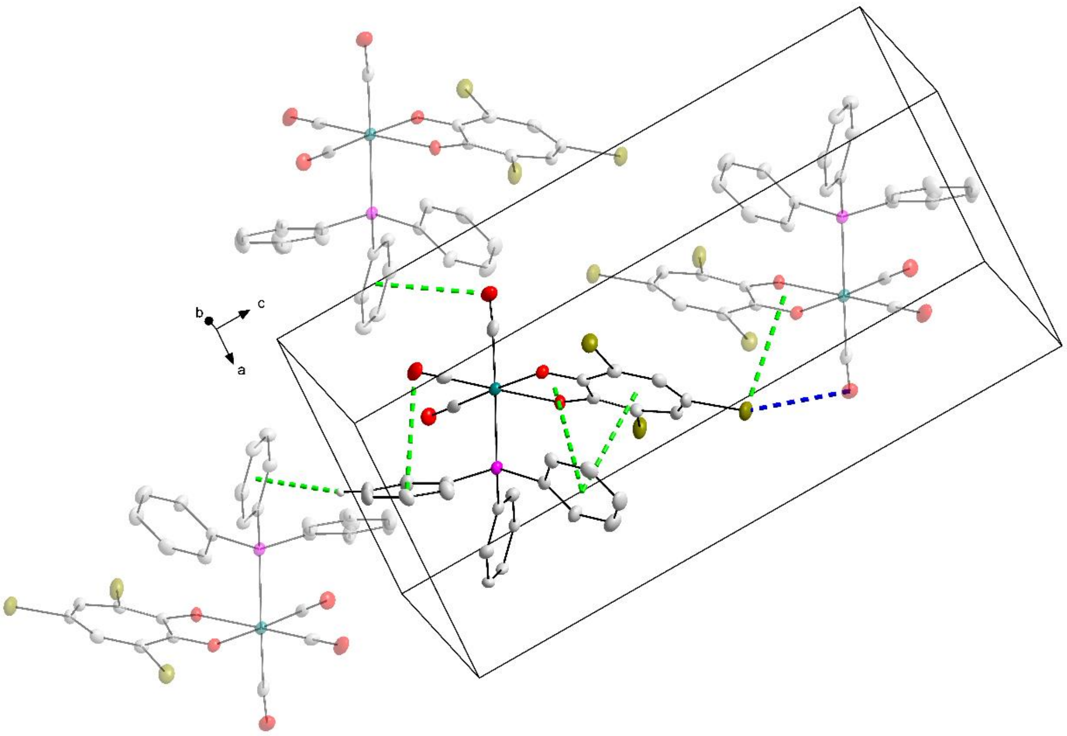

Table S4 Summary of the π-interactions and the short contacts observed in the structure of **2**.

	H/Br/O/Cg···Cg/O/Br (Å)	C—H/Br/O···Cg (°)
C23—H23···Cg3^{i}	2.66	160
C15—Br2···Cg1^{ii}	3.7414(15)	93.90(13)
C2—O2···Cg2	3.626(4)	88.9(3)
C3—O3···Cg3^{iii}	3.438(4)	100.5(3)
Cg1···Cg4	3.865(2)	
Cg4···Cg5	3.690(2)	
Br2···O3^{ii}	3.250(4)	
Br1···Br3^{iv}	3.4809(7)	
Br3···Br1^{v}	3.4809(7)	

Cg1 = centroid of Re1,O11,C11,C12,O12; Cg2 = centroid of C21-C26; Cg3 = centroid of C31-C36; Cg4 = centroid of C41-C46; Cg5 = centroid of C11-C17. Symmetry codes: (i) 1-x,1-y,-z, (ii) 1-x,1-y,1-z, (iii) -1+x,y,z, (iv) -1+x,-1+y,z, (v) 1+x,1+y,z.

Figure S5 Illustration of the one-dimensional chain formed between Br1 and Br3 (-1+x,-1+y,z) and Br3 and Br1 (1+x,1+y,z) in the structure of **2**. Hydrogen atoms omitted for clarity.

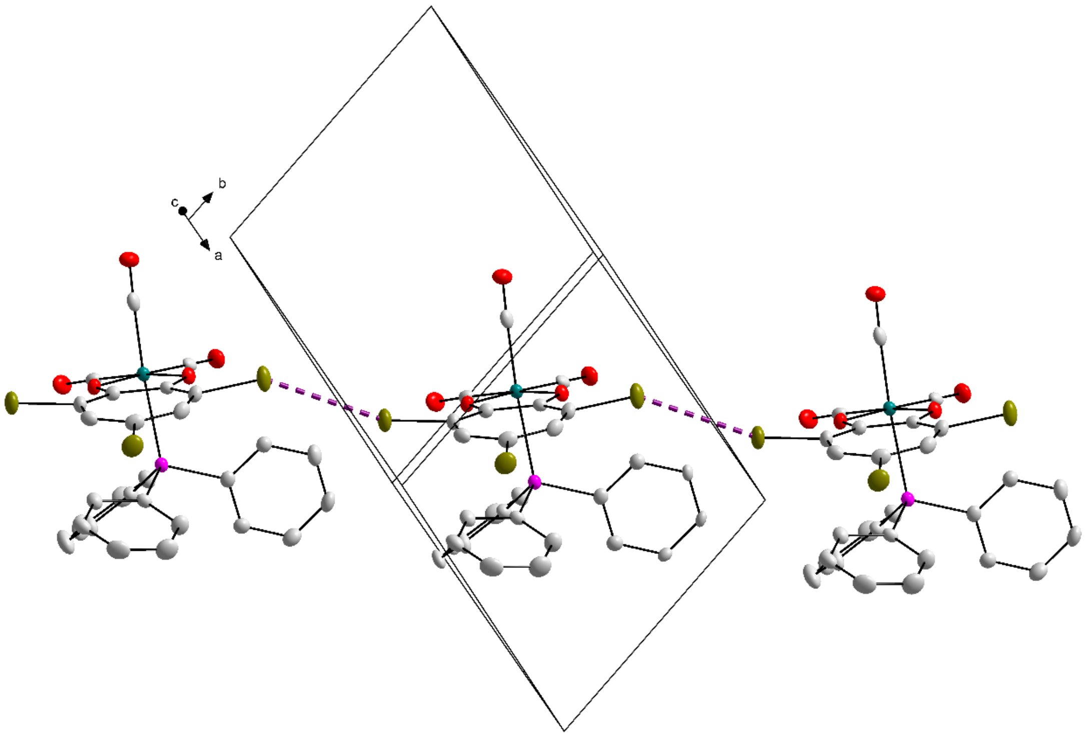

Figure S6 Illustration of the dihedral angle observed between the plane through Re1,C1,O1,C2,O2 and the plane through O11,O12,C11-C17 for **1** and **2**.

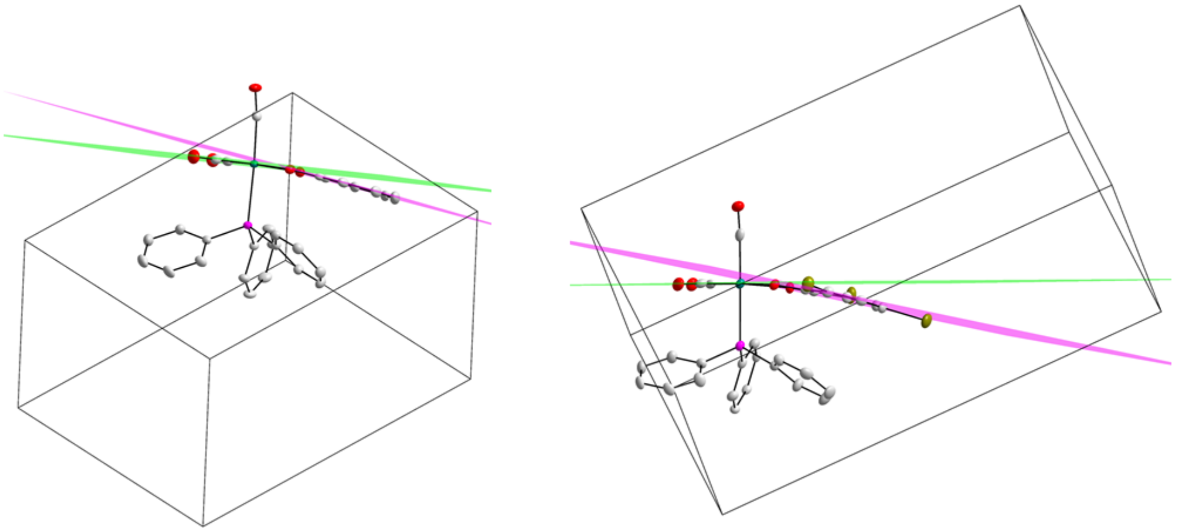

1 **2**
